# Cellular Chaperone Function of Intrinsically Disordered Dehydrin ERD14

**DOI:** 10.3390/ijms22126190

**Published:** 2021-06-08

**Authors:** Nikoletta Murvai, Lajos Kalmar, Beata Szabo, Eva Schad, András Micsonai, József Kardos, László Buday, Kyou-Hoon Han, Peter Tompa, Agnes Tantos

**Affiliations:** 1 Research Centre for Natural Sciences, Institute of Enzymology, 1117 Budapest, Hungary; murvai.nikoletta@ttk.hu (N.M.); lk397@cam.ac.uk (L.K.); szabo.beata@ttk.hu (B.S.); schad.eva@ttk.hu (E.S.); buday.laszlo@ttk.hu (L.B.); Peter.Tompa@vub.be (P.T.); 2 Department of Biochemistry, Institute of Biology, ELTE Eötvös Loránd University, 1117 Budapest, Hungary; 3 Department of Veterinary Medicine, University of Cambridge, Cambridge CB3 0ES, UK; 4 ELTE NAP Neuroimmunology Research Group, Department of Biochemistry, Institute of Biology, Eötvös Loránd University, 1117 Budapest, Hungary; micsonai@ttk.elte.hu (A.M.); kardos@elte.hu (J.K.); 5 Biomedical Translational Research Center, Division of Convergent Biomedical Research, Korea Research Institute of Bioscience and Biotechnology, Daejeon 34141, Korea; yoonje55@naver.com; 6 Gene Editing Research Center, Division of Convergent Biomedical Research, Korea Research Institute of Bioscience and Biotechnology, Daejeon 34141, Korea; 7 VIB-VUB Center for Structural Biology (CSB), Vlaams Instituut voor Biotechnologie (VIB), 1050 Brussels, Belgium; 8 Structural Biology Brussels (SBB), Vrije Universiteit Brussel (VUB), 1050 Brussels, Belgium

**Keywords:** intrinsic structural disorder, plant chaperone, heat stress, structure-function relationship, dehydrin, early response to dehydration, LEA protein, CD spectroscopy

## Abstract

Disordered plant chaperones play key roles in helping plants survive in harsh conditions, and they are indispensable for seeds to remain viable. Aside from well-known and thoroughly characterized globular chaperone proteins, there are a number of intrinsically disordered proteins (IDPs) that can also serve as highly effective protecting agents in the cells. One of the largest groups of disordered chaperones is the group of dehydrins, proteins that are expressed at high levels under different abiotic stress conditions, such as drought, high temperature, or osmotic stress. Dehydrins are characterized by the presence of different conserved sequence motifs that also serve as the basis for their categorization. Despite their accepted importance, the exact role and relevance of the conserved regions have not yet been formally addressed. Here, we explored the involvement of each conserved segment in the protective function of the intrinsically disordered stress protein (IDSP) *A. thaliana’s* Early Response to Dehydration (ERD14). We show that segments that are directly involved in partner binding, and others that are not, are equally necessary for proper function and that cellular protection emerges from the balanced interplay of different regions of ERD14.

## 1. Introduction

Chaperone proteins have developed in evolution for the protection of cells against a variety of abiotic stresses, including drought, high or low temperature, osmotic stress, different salts, or heavy metals. These protective proteins come in a variety of shapes and forms as they need to be able to function under unfavorable conditions and on a plethora of different clients. One of the largest groups of chaperones are heat shock proteins (HSPs), originally identified as being expressed under conditions of elevated temperatures. These are complex globular proteins that come in a variety of sizes and utilize intricate molecular structures and interaction networks to fulfil their functions [1]. Nevertheless, not only globular proteins can serve as chaperones, as a growing body of information underlines the importance and efficiency of a chaperone class encompassing disordered proteins [2,3].

Dehydrins, which are categorized as Group 2 LEA (Late Embryogenesis Abundant) proteins, are such disordered chaperones that function in an ATP-independent manner [2]. Proteins in this family have multifunctional roles in stress response in many taxa [4]. They are generally characterized by high net charge and the presence of polar amino acids; they are enriched in glycine and lysine residues but are depleted in cysteine and tryptophan [5], suggesting a disordered character. Their categorization is based on the highly conserved segments that can be found in their sequences [6] in different combinations: (i) The K-segment is an approximately 15 amino acid long conserved lysine-rich motif present in all cold-expressed dehydrins. It can be found in multiple copies (1–12) within a dehydrin and, according to in silico predictions, it tends to adopt an amphipathic α-helix structure [7,8]. Presumably, K-segments can play a role in shaping the interaction with partner molecules, which is supported by our earlier observations [9]. (ii) The S-segment is a serine-rich region that can be phosphorylated, and, in most examples, it is wedged between the K-segments. The exact function of its phosphorylation is not yet known, but it may play a role in the protein’s delivery to the nucleus or in membrane stabilization. (iii) The Y-segment is a conserved region at the N-terminus of dehydrins that shows homology to the nucleotide binding regions of plant proteins and bacterial chaperone proteins. (iv) The ChP-segment is a highly charged, lysine-rich region that bears a strong resemblance to the charged linker region of the Hsp-90 chaperone protein found in animals. (v) The q-segment is a less conserved, apolar region composed of glycine or proline and alanine amino acids, usually located between two K-segments [6,10].

The *Arabidopsis thaliana* genome encodes for 10 different dehydrins that probably have partially overlapping functions [11,12]. ERD14 is one of the *A. thaliana* dehydrins that belongs to the SK2 dehydrins subgroup, and it contains an additional hydrophobic region (H region) in its sequence. Its expression is induced under different stress conditions, such as heat and osmotic stress, and it can also be found in high quantities in rapidly dividing tissues, such as meristems [13]. In vitro and in vivo studies have indicated that ERD14 is a potent chaperone for a number of substrates [14,15]. Experiments with lysozyme, alcohol dehydrogenase, luciferase, and citrate synthase model substrates have shown that this protein is capable of preventing heat-induced loss of activity and subsequent aggregation of the enzymes [3]. They did not show, however, a chaperone activity in reactivating denatured proteins, and the exact mechanism of its chaperone function is yet unknown.

Conserved segments in ERD14 create a specific pattern of charge- and structure-distribution that may be crucial for the proper chaperone function of the protein.

**Figure 1:**
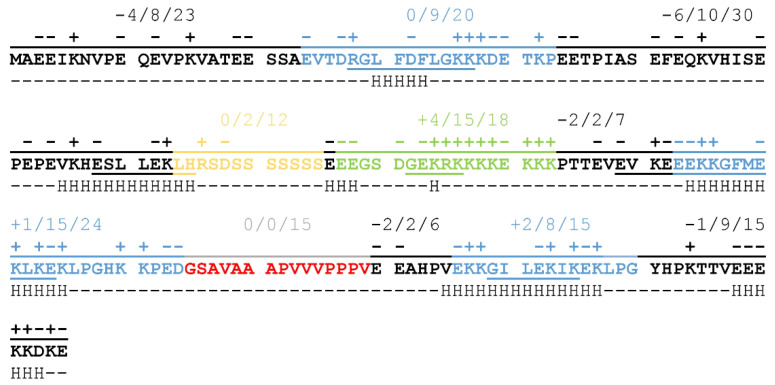
Conserved segments, charge distribution, in silico, and experimental α-helical propensities of the ERD14 protein. Conserved regions: Ka-, Kb-, Kc- (blue), S- (yellow), Chp- (green), and H-segment (red) indicated in the sequence of ERD14. In addition to individual charges above the regions, net charge/number of charged side chains/length of regions summed up. Underlined letters show residues with α-helical propensity calculated from nuclear magnetic resonance (NMR) chemical shifts [9,16] by δ2D method [17]. Below the residues, H indicates the α-helical propensity estimated by in silico PredictProtein analysis [18,19,20].

The balanced distribution of charged amino acids throughout the full length of the protein results in a sequence that has a low overall negative charge (−9 at neutral pH resulting from 46 negatively and 37 positively charged sidechains) carrying a significant portion of charged regions (Figure 1). The N-terminal half of the protein is negatively charged, with two non-conserved, disordered regions of strong negative charge, while the C-terminal region partly compensates for this, mainly through the region of the Chp-segment consisting of 15 charged residues out of 18 (in green on Figure 1). K-segments (blue on Figure 1) achieve neutral net charges by alternating positively and negatively charged residues, a pattern that might be important in partner binding [9]. The S-segment (yellow on Figure 1), on the other hand, consists mostly of polar, non-charged amino acids, whose exact relevance is not yet clear, although phosphorylation of the serine sidechains may have a regulatory function. The H-segment (red on Figure 1) also lacks charged residues, but it is highly hydrophobic and contains several proline residues. This may be important for the recognition of exposed hydrophobic patches on partner proteins while also helping to keep ERD14 in a disordered structural state.

In our previous work, we identified several different *E. coli* proteins bound by ERD14, supporting the idea of its promiscuous protein binding and protection. We provided evidence that ERD14 is localized in the cytoplasm during heat stress, arguing against its functioning through membrane protection [9]. Our in-cell NMR measurements also confirmed that the conserved K-segments that sample helical structural states in vitro [16] are the ones that participate mainly in molecular interactions, while most of the other regions remain disordered. Nevertheless, being able to bind proteins is not the only prerequisite of chaperones, highlighting the importance of the regions outside of K-segments in the function of ERD14.

In order to gain deeper insight into the molecular architecture needed for the protective function of ERD14, we designed several mutants by deleting one or more of the conserved regions (1x: ∆Ka, ∆Kb, ∆Kc, ∆Chp, ∆H, ∆S; 2x: ∆Kab, ∆Kbc, ∆Kac; 3x: ∆Kabc) or prepared scrambled mutant sequences (Full-Scr, Scr-KabcS, Scr-Kabc, ScrKcS, Scr-Kc, Scr-S) and determined the heat-resistance of *E. coli* cells, expressing the different mutants and studying the structural consequences of the deletions or sequence modifications.

## 2. Results

ERD14 is a typical representative of the SK2 group that harbors several potentially important sequential features. In addition to its unique charge-distribution, secondary structural elements are also dispersed along the sequence of ERD14. As indicated on Figure 1, short helical regions can be found mostly in the K-segments, but a non-conserved region at the border of the S-segment, as well as the ChP-segment, samples helical conformations in vitro, as suggested by NMR spectroscopy [16]. The observed patterning of charges and secondary structural elements suggest that the different regions have specific functions and possibly cooperate with each other to achieve biological function.

**Figure 2:**
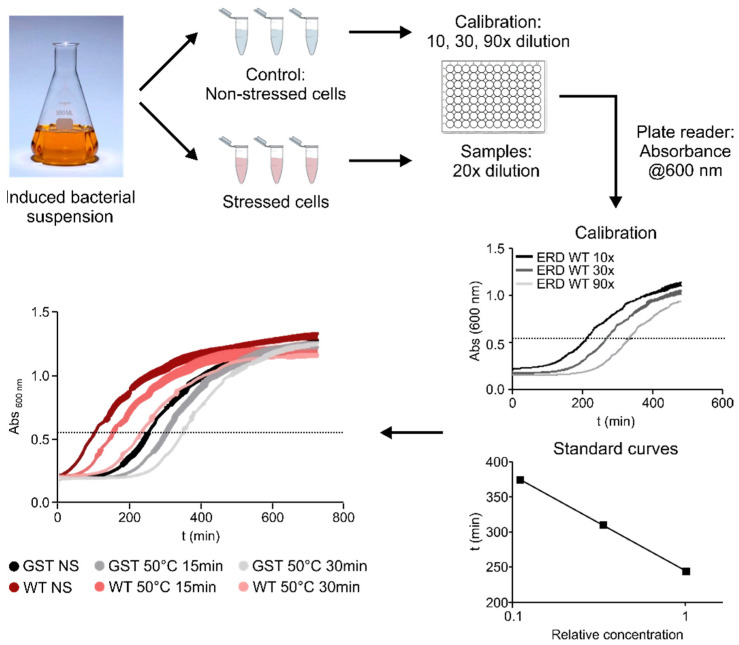
Overview of the viability assay. After a standard induction period, cells were stressed by a heat shock of 50 °C for 15 min (final set-up, after optimization of the stress conditions by screening at different temperatures for different time length shown at the left bottom panel). Viability of stressed and non-stressed samples were compared based on their cell growth after appropriate dilution in fresh medium (Calibration—grey curves). Cell growth was followed by absorbance at 600 nm. Half-time of growth curves varies linearly, with dilution plotted on a logarithmic scale, i.e., it is informative of the concentration of viable cells in the sample. For our experiments, we have set the OD at 0.6 as this period of growth by convention and applied serial dilutions to obtain the calibration curve and calculate the relative survival ratios of the different samples (NS: non-stressed samples, S: stressed samples). Based on this, we can compare the viability of cells exposed to the same stress conditions but expressing various constructs (colored curves).

**Figure 3:**
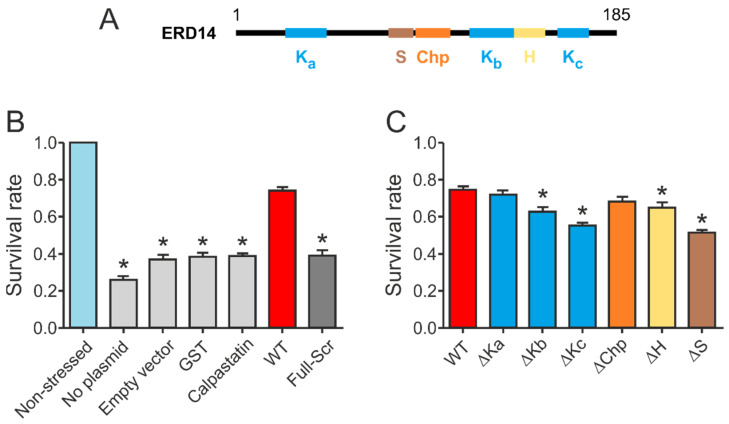
Conserved regions of ERD14 contribute to cell protection. (**A**) Schematic representation of the conserved regions within ERD14. (**B**) Survival rate (viability) is reduced by the applied heat stress to 26.2% (compared to non-stressed cells) for non-transformed cells, 36.8% for cells containing an empty vector, and about 39% in the case of overexpression of control proteins: GST (38.4%) and IDP calpastatin (38.7%). Overexpression of ERD14 increases the survival rate to 74.5%. Removing the conserved regions by randomizing the amino acid sequence (Full-Scr) abolished the protective effect (viability 38.9%). (**C**) Protective effect of a series of mutants in which conserved regions of the protein have been individually deleted. Data represent mean ± SEM and the results of at least 18 parallels for each construct. Significant differences (*p* < 0.05) compared to WT ERD14 are labeled with asterisks (*).

To assess the contribution of different regions of ERD14 to the protective effect of the protein, we developed a system using a heat-treatment that reduces the viability of *E. coli* cells to around 25% of the original value (Figure 2 and Figure 3B). After testing various conditions (Figure 2), we chose a treatment of 50 °C for 15 min, as this gave the optimal difference between the stressed and non-stressed cells, allowing a wide dynamic range to detect differences in survival rates. The protective effect of each ERD14 variant was assessed as the difference of the survival rate (DSR) of the cells compared to the ones expressing the wild-type protein. This system has already proved to be useful for the determination of the in vivo structural state and the molecular partners of ERD14 [9], and it has several beneficial aspects for our purposes. Most importantly, it enabled us to investigate several different protein constructs in a timely manner and, by eliminating the redundancy of plant chaperones [12], it largely simplified the interpretation of the results. Because dehydrins are known to have broad substrate specificity [21,22,23], and we were able to identify several partner proteins of ERD14 in *E. coli* cells, we found this system to be an appropriate model system for our study.

Our initial results confirmed that wild type ERD14 is capable of protecting cells against the applied heat stress, increasing their survival rate to 70% (Figure 3B). None of the control proteins were capable of such protection, and induction of an empty expression vector did not affect stress resistance either. The importance of specific sequential components (Figure 3A) in the function of the protein was suggested by the fact that a variant with similar amino acid composition but randomized sequence (Full-Scr) did not protect the *E. coli* cells against heat stress (Figure 3B).

Our structural studies indicated that the conserved K-segments were primarily involved in direct partner binding within the cells [9], prompting us to design a series of deletion mutants targeting these regions (for constructs, cf. Supplementary Table S1). Even though the other conserved ERD14 regions, the S-, Chp-, and H-segments, did not appear to play major roles in partner recognition, we have also created deletions of these as well to determine if they contributed to the chaperone effect. The constructs were expressed in *E. coli* (at levels comparable to that of WT ERD14, cf. Supplementary Figure S1), and their protective effects in the cell viability assay were measured (Figure 2).

Deletion of different conserved regions affected the chaperone activity of ERD14 to different extents (Figure 3C). Of the three K-segments, removal of Kc (∆Kc) impaired activity the most, reducing the remaining cell viability from 74.5% (wild type) to 54.9% (DSR: 19.6%). The observed level of protection was always normalized to the actual expression level of the given construct, assuming linearity of response (cf. Experimental Procedures and Supplementary Figure S2). Survival rates changed linearly with increasing ERD14 concentrations for the wild type and the ∆Kc mutant ERD14 when we applied an IPTG-inducible system, as well as in an arabinose-inducible set-up. The latter enabled us to avoid the side effects of changing cellular density during elongated expression times. The reduction in protection by deleting the S-segment was the most pronounced (DSR 23.4%), and it is commensurable with that of Kc. The deletion of Kb (DSR 11.9%), H (DSR 9.8%), and Chp (DSR 6.6%) have intermediate effects, whereas deletion of Ka (DSR 3%) causes almost no change in activity. These results suggest that Kc and S are the primary sites of activity, together with varying contributions of other segments. These notions were further confirmed by the in vitro chaperone activities of these mutants (Supplementary Figure S3 and Table S4). In these experiments, the ∆Kc and ∆S variants showed similarly reduced chaperone activities as the FS ERD14 variant. This is an interesting observation, especially in light of the fact that all K-segments participate in partner binding within a cell, whereas the S-segment does not [9]. Although partner binding in itself is a property of large amounts of proteins, only a small portion of them exhibit chaperone activity. Beyond the necessary partner binding regions, a chaperone is expected to have segments that contribute to or are responsible for chaperone function, such as the S-segment.

**Figure 4:**
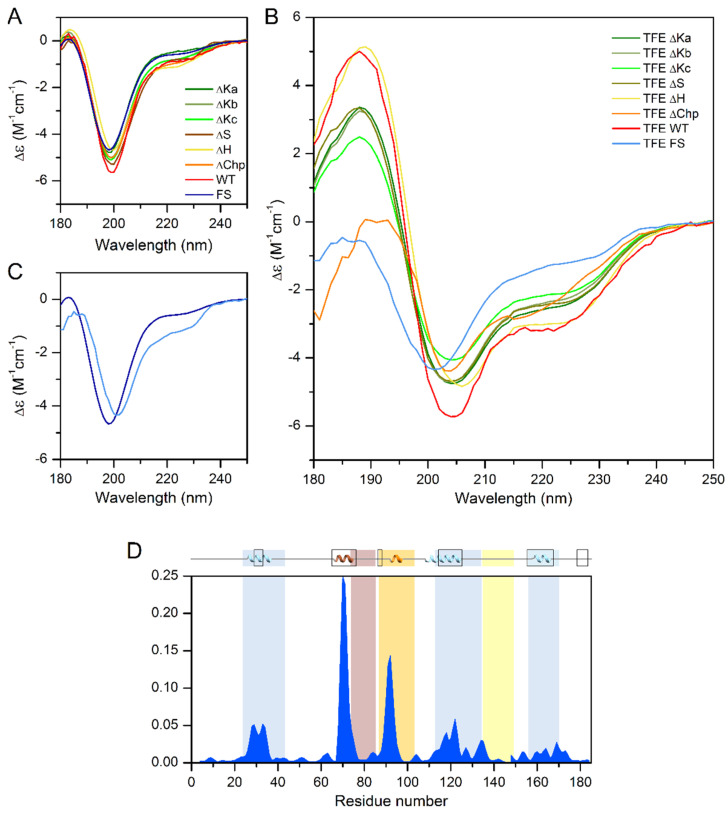
CD measurements of single deletion mutants and Full-Scr construct of ERD14 with TFE induced secondary structure changes. (**A**) Circular dichroism spectra of the ∆Ka-, ∆Kb-, ∆Kc-, ∆S-, ∆H-, ∆Chp-, and Full-Scr (FS) constructs of ERD14, recorded in 10 mM Na-phosphate buffer (pH 7.4), indicating similar, mainly disordered, structures, which is demonstrated by a characteristic minimum below 200 nm and low amplitude around 220 nm. (**B**) CD spectra of the different constructs of ERD14 recorded in Na-phosphate buffer containing 30% TFE. There are significant differences in the extent of the induced secondary structure, as shown by the spectral differences between the variants. (**C**) Comparison of the CD spectra of the FS ERD14 variant in native (dark blue) and in 30% TFE containing (light blue) buffer, indicating minor effects of TFE on the structure of the variant with scrambled sequence. (**D**) The degrees of formation of α-helices (blue areas) obtained with the δ2D method [17] using Nuclear Magnetic Resonance Chemical shifts mapped to the WT ERD14 sequence, and its graphical representation inserted above the diagram shows good accordance with the in-silico estimated α-helix content [20] (boxes at the top).

Because the ChP-segment and the three K-segments tend to adopt α-helical conformations, as detected by NMR [9,16], we compared the helix content of the different single deletion mutants by CD spectroscopy. In phosphate buffer, all tested proteins showed a CD spectral shape characteristic of a disordered protein (Figure 4A), very similar to that of the wild-type ERD14. Because CD spectroscopy reflects the average secondary structure content, this indicates that the free ERD14 molecules are essentially disordered, and the helices shown by NMR [9] are truly only transient and might be stabilized by interacting with partner molecules. The inherent tendency of the wild-type protein and the deletion mutants to form α-helical structures was confirmed by testing their structure in the presence of the helix-inducing trifluoro-ethanol (TFE). The addition of 30% TFE increased the helicity of the wild-type protein from 5% to 25% (Figure 4B). Deletion of the K segments reduced the helicity in TFE to 16–21% (21%, 20%, and 16% for ∆Ka, b, and c, respectively), indicating that the overall structure of the protein was probably not perturbed in these mutants. Similar observations were made for the S-segment, which had a residual helicity of 20% in TFE. Removal of the H-segment resulted in a minor change in helix-forming tendency (23% helix content in TFE), and the ∆Chp variant also showed a somewhat reduced helix-forming tendency with 22% helix content. In contrast to the single deletion mutants, the Full-Scr ERD14 variant remained almost completely disordered, with only 9% helix content even in the presence of TFE (Figure 4C,D). Intriguingly, the loss of helicity by deletion of the individual segments sums up significantly more than the overall helix content of WT ERD14 (67 residues vs. 46, respectively), which indicates that the structure-forming tendencies of the segments are not independent, nor additive, and there might be an interplay between ERD segments.

Table 1 shows the changes in the number of residues that are in α-helical conformation in the presence of TFE in the single deletion mutants, compared to the wild-type protein, thus we can gain valuable insight into the helix-forming tendencies of each conserved segment.

Intriguingly, the PredictProtein online bioinformatics structure analysis tool ([20]) predicts α-helix for 50 residues in the ERD14 sequence, which is far more than experimentally observed by CD under native conditions (9 residues); however, the prediction fits well to the CD analysis of the TFE-induced structure (46 residues in α-helix). NMR measurements are also in good correlation with this, as 44 residues show transient helical conformations in vitro [16]. These are not stable enough for the CD to pick up in phosphate buffer, but as we can see, they can be stabilized and measured by the addition of TFE. PredictProtein assigns all three K-segments as helical; however, similarly to CD (Table 1), it predicts the lowest helicity for Ka. It also indicates some helicity for Chp and S and for regions that are located outside of the conserved segments. Apart from three residues located sporadically in β-sheet, the rest of ERD14 is predicted to be disordered. These bioinformatics results are partly fit to the experimental ones of CD and the earlier NMR, and while being useful, should be handled with caution.

If we compare the structural effects observed by CD spectroscopy with the changes in the survival rates of bacteria expressing different constructs (Table 1 and Table S3), we can draw interesting conclusions regarding the structure-function relations of ERD14. While the structural changes induced by removing each of the K-segments were substantial, Kc causing the highest, their effect on the activity of the protein differed more significantly. While the deletion of Kc caused the highest activity decrease, Ka, despite its helix contribution, had almost no effect on activity. Conversely, the ∆H mutant was a significantly less effective chaperone than the WT protein, even though the change in α-helical content was the lowest (Figure 3B and Table 1). The deletion of the ChP-segment resulted in a moderate reduction of helicity, while it did not significantly affect the protective activity. Finally, removing all structural tendencies and sequence motifs by randomizing the full sequence of the protein results in a complete loss of activity (Figure 3A and Table 1).

Taken together, these observations suggest that secondary structure formation is a necessary, but not sufficient, prerequisite of the protective effect of ERD14, as partner binding and a detectable redundancy of the regions also play important roles.

**Figure 5:**
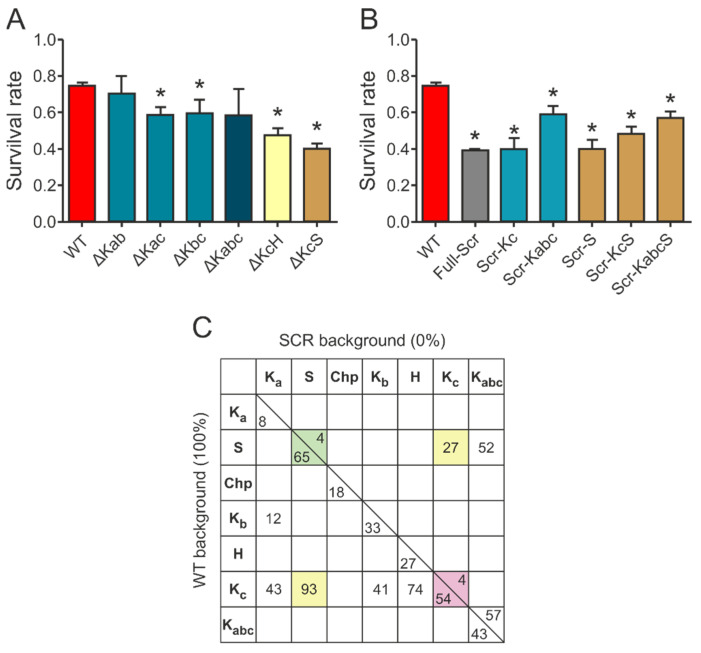
Interplay of different conserved elements in ERD14. (**A**) Protective effect of double- and triple deletion mutants, in which two or three segments are deleted (K-segments Ka, Kb, and Kc, as well as Chp-, S-, and H-segments; cf. sequence: Supplementary Table S1). Data represent mean ± SEM and the results of at least 18 parallels for each construct. Significant differences (*p* < 0.05) compared to WT ERD14 are labeled with asterisks (*). (**B**) Protective effect of scrambled mutants, in which either the full sequence is scrambled (Full-Scr), or particular binding regions are kept intact (e.g., Kc in Scr-Kc) and the rest of the sequence is randomized. Data represent mean ± SEM and the results of at least 18 parallels for each construct. Significant differences (*p* < 0.05) compared to WT ERD14 are labeled with asterisks (*). (**C**) The relative % contributions of motifs of ERD14 (Ka, S, Chp, Kb, H, and Kc) and their combinations (e.g., KcS is combined Kc and S) are quantified, as given in Materials and Methods (Supplementary Table S2). Their % contributions to the activity above the given background, WT ERD14 and Full-Scr ERD14, either alone or in combinations, are presented in a matrix. Numbers highlighted in color demonstrate that effects are usually higher in the WT than in the scrambled background.

Based on the results of the survival assays and the CD measurements, we concluded that there is a significant redundancy of the motifs that manifests at several levels in the apparent exchangeability of individual motifs. This is shown by deleting multiple motifs (Figure 5A). For example, the chaperone activity of the deletion mutant ∆Kc is not further reduced by the deletion of the Ka or Kb segment (cf. ∆Kac: DSR 15.7%, ∆Kbc: DSR 14.7%, and ∆Kabc: DSR 15.6%), i.e., the Ka and Kb segments are rather redundant and, without the more effective Kc or S-segment, are of little additional effect. Furthermore, ∆Kab (DSR 4.2%) is almost as effective a chaperone as WT ERD14, i.e., even Ka and Kb combined have little protective function. Surprisingly, the single deletion of Kb caused more significant reduction (DSR 11.9%), suggesting that there might be a complex interplay between the segments. Combined deletion of the Kc and S-segments renders the protein almost completely ineffective (∆KcS: DSR 34.1% vs. Full-Scr: DSR 35.6%), while deletion of the H-segment together with the Kc increases the activity loss of either of the segments deleted alone (∆KcH: DSR 26.8% vs. ∆Kc: DSR 19.5% and ∆H: DSR 9.8%).

In an attempt to gain a deeper insight into the functional interplay of the different conserved segments, we created a further variety of mutants in which different elements of ERD14 were modified, either individually or in various combinations (cf. Supplementary Table S1). First, we have investigated if any of the effective motifs, combined with the rest of the proteins, scrambled (kept disordered but without any recognizable sequence motifs), has a protective effect (Figure 5B). Keeping any of the most effective segments (Scr-Kc or Scr-S) results in a protein with negligible chaperone activity, similar to the entirely scrambled protein (Full-Scr). Keeping two segments (Scr-KcS) results in a more active protein, whereas keeping three (Scr-Kabc) or four (Scr-KabcS) makes it an even more potent chaperone. The importance of the other sequential elements is underlined by the fact that keeping even four segments intact cannot completely rescue the chaperone activity of the protein.

These results support a mechanism where binding at multiple points is complemented by the functional role of a disordered linker and/or terminal regions of ERD14.

Our previous observations [9,14,16], combined with the results detailed above, suggest that ERD14 functions by an interplay of transient partner binding via short binding motifs and space filling by significant residual disorder in the linker and flanking regions. To gain a deeper insight into the structure-function relationship of the protective effect of ERD14, we devised a strategy to quantitate and compare the contribution of different segments of the protein to its in vivo activity. We found that scrambled ERD14 has no activity (0%) and have taken the activity of WT ERD14 (under the given conditions) as 100%. We then argued that, for a deletion construct in the WT background, the addition of the given motif would restore activity to 100%, i.e., its contribution corresponds to the reduction of activity upon its deletion. For scrambled mutants, we argue that the addition of the given region to the inactive scrambled construct (Full-Scr) raises activity from 0% to the level measured, i.e., the contribution of the segment in the Scr background corresponds to the activity measured for the given construct. The numbers thus calculated (Supplementary Table S2 and Figure 5C) show that the two numbers for the same region (i.e., for WT and Scr background) are different, which points to a strong synergy between different, conserved, and linker regions of the protein and allows several mechanistic conclusions.

## 3. Discussion

### 3.1. The Role of K Segments

Our results point to the importance of short segments of ERD14 in its protective function, in particular the K-segments Kb and Kc, and the H and S motifs. There have been multiple prior links of evidence for the importance of K-segments in dehydrins. First of all, these and the S-segment are the most conserved regions in dehydrins. Dehydrins are actually defined and classified by the architecture of these segments [24,25], e.g., ERD14 is classified into the SK2 family, primarily because Ka has little similarity to the canonical K-segment. In vitro studies have shown that deletion of K-segments of wheat dehydrin DHN-5 renders the protein inactive [26], whereas sequential removal of K-segments from ERD10 causes a gradual decrease in its protective activity [27]. Interestingly, in 8 out of 10 dehydrins from *A. thaliana*, K-segments occur more than once, up to 6 K-segments in protein XERO2 [24], which is a strong evolutionary argument for the operation of a chaperone model relying on multivalent binding.

Whereas the interaction of K-segments with partners via motifs follows from our results and many prior observations [9,28], the exact mode of interaction with client proteins remains an open question. The detailed nature of interaction cannot be ascertained from in vivo analyses, but detailed studies of traditional chaperones suggest that hydrophobic interactions must play an important role [29]. The H-segment is hydrophobic, whereas K-segments constitute amphipathic helices with a hydrophobic face [30]. Prior to stress, they probably use their charged face to bind to the surface of native proteins, and when protein clients start to unfold under stress, a switch to their hydrophobic face can be surmised. In all, induced folding is probably an important element of partner recognition by binding motifs of ERD14 [9].

### 3.2. Synergy and Redundancy of ERD14 Segments

From analyzing and comparing the relative contribution of the various segments to cellular protective function, more detailed insight can be gained. (i) The function of ERD14 is distributed in the sequence, i.e., practically all regions of the disordered chaperone contribute to its function (there is no 0 along the diagonal on Figure 5C). That is, unlike with folded proteins, enzymes, or classical chaperones, there is not one particular region whose deletion abolishes activity, while the binding of any region alone is not sufficient for cellular protection. (ii) Different regions carry different portions of function: S and Kc being the strongest, and Ka the weakest. (iii) Distribution also comes from the fact that more regions always contribute more to the effect; in WT background: Kac > Ka, KaS > S, and Kc and Kbc > Kb, whereas, in the Scr background, KcS > Kc and S. (iv) The numbers also infer synergy with further regions of the protein. That is, both Kc and S (and the two combined, i.e., KcS) have much stronger contribution in the WT than in scrambled background, i.e., they functionally synergize with further motifs/regions. This appears to be primarily the H-segment, as Ka or Kb do not add much to the contribution of Kc. In fact, if we sum the contribution of all the individual motifs (numbers along the diagonal, on the WT background side), they add up to about 200%, meaning they do not act independently of each other. (v) This notion of interdependent activity of the different conserved regions is further corroborated by our structural studies. The effect of deletion of K-segments caused a significant decrease in helicity. While Kc exhibited the highest effect, similarly to its highest protective effect among K-segments, the decrease in helicity is not proportional to the loss in the protective effect, especially for Ka. The removal of the otherwise hydrophobic H region results in the lowest change in helicity; however, it has a significant protective effect. Removal of the polar but weakly charged S-segment significantly decreases the ability of the protein to form helices, even if NMR and bioinformatics do not assign much helix for this region, indicating its possible structure-stabilizing role on the other segments of ERD14. The fact that the structural changes induced by deletion of the individual segments does not lead to a similar level of activity loss suggests that secondary structure is not the only premise of activity in the case of ERD14. (vi) As Kc (a region binding to partners) and S (a region that remains disordered) [9] dominate the protective effects, this points to the functional interplay of binding and disordered regions in the cellular chaperone function of ERD14. (vii) With regards to the functionally important and conserved S-segment [24], it remains unbound and disordered in our system [9], thus it probably contributes to increasing the solvation potential of ERD14. Further, as dehydrins—among them ERD14—are known to be regulated by phosphorylation [31,32], ERD14 may be most effectively regulated by phosphorylating serines in the S-segment. (viii) Taking into account the differences in the density of charged residues, net charge, hydrophobicity, and amino acid composition of the various regions of ERD14, and comparing it to the observed protective effects and secondary structure tendencies, it seems obvious that they contribute to the chaperone activity in different aspects in a synergistic manner, including the transient binding of a variety of partner molecules, keeping the solubility of the complex, stabilizing the folding of the partner, and protecting it against aggregation.

## 4. Materials and Methods

### 4.1. Cell Viability Assays

Cell viability was measured in the LB medium, by observing the time it takes for cells to reach an optical density of 0.6 at 600 nm of appropriately diluted samples. To this end, BL21 Star (DE3) *E. coli* cells, overexpressing one of various ERD14 constructs or control proteins (GST, calpastatin (CAST)), were grown in 5 mL LB medium containing 50 µg/mL carbenicillin overnight at 37 °C under continuous shaking at 200 rpm. The next day, 50 µL aliquots were transferred into 5 mL LB medium containing 50 µg/mL carbenicillin and were grown for 2 h. Protein expression was induced with 0.5 mM IPTG for 3 h; 0.5 mL aliquots were taken and either stressed or not stressed before appropriate dilution into LB medium into the wells of a 96-well plate. Measurements of absorbance at 600 nm were taken every 2 min in a BioTek Synergy Mx microplate reader for 12 h at 37 °C by 200 rpm shaking; viability was associated with the time taken for the cells to reach an optical density of 0.6, roughly the half-maximal value they attain after long incubation periods (cf. Figure 2). From a number of different conditions, we selected 50 °C × 15 min for an optimal stress effect—as defined by a survival rate of the untransformed cells between 20% and 30%. This provides a wide dynamic range to assess the protective effects of the different overexpressed protein constructs. The observed protection effect was normalized to the actual expression level of the given construct in the given experiment, enabled by the linearity of dependence (cf. Supplementary Figure S2). In short, the actual concentration was determined, and the expected protection at a concentration matching that of WT ERD14 was calculated by linear intra- or extrapolation. Further details, such as optimization of the assay, and varying concentration of constructs, are given in Supplementary Information.

### 4.2. Constructs, Recombinant Proteins, Mutagenesis

Various deletion constructs of ERD14 were generated from the wild-type sequence by PCR. Scrambled sequences (Full-Scr, Scr-Kc, Scr-S, Scr-SKc, Scr-Kabc, and Scr-KabcS), designed in silico, were generated by total cDNA synthesis. For in-cell stability determination, ERD14 was FLAG-tagged by inserting it into the expression vector pT7-FLAG 2. Sequences of all constructs and the control proteins are delineated in Supplementary Table S1, whereas construction details can be found in Supplementary Information.

Differential survival rate (DSR) was calculated as follows: percentage of cells expressing specific mutant constructs that survived the applied heat stress was extracted from the percentage of survival when the WT ERD14 was expressed, i.e., if the survival rate was 80% for the WT and 50% for a mutant, then the DSR of the specific mutant is 30%. This means that the larger the DSR, the less efficient a chaperone the mutant is.

### 4.3. Scrambled ERD Mutants

We used a multistep approach during the design of the scrambled ERD14 mutants: (i) we generated 10,000 random sequences by using the amino acid composition of ERD14, keeping the sequence set non redundant during the process; (ii) we used IUPred [33] (long disorder prediction) to predict disorder propensity on all sequences and filtered out those that were significantly different from the disorder characteristics of the original ERD14 (different average disorder score or different disorder score variance); (iii) as the main reason for working with scrambled mutants was to test the effect of the amino acid composition only, we used the ANCHOR algorithm [34] to predict potential interaction regions and exclude sequences with high confidence interaction regions; (iv) as the randomized sequences turned out to be highly prone to generating regions with high coiled-coil propensity, we introduced a further filtering step: we used the COILS server [35] to predict coiled-coil regions and exclude sequences with high coiled-coil propensity; (v) as a final step, we used a charge distribution plot on the selected candidates (https://www.bioinformatics.nl/cgi-bin/emboss/charge (accessed on various dates in October 2016 and February 2017) to visually filter out sequences with uneven charge distribution. A more detailed description of the selection and post-filtering can be found in the Supplementary Methods.

### 4.4. Expression and Purification of Recombinant Proteins

The pET-22b (+) vector containing the appropriate construct was transformed into competent *E. coli* BL21 * (DE3) pLysS cells and grown in LB medium containing 0.05 mg/mL carbenicillin overnight at 37 °C with shaking at 200 rpm. After inoculation with the starter cell culture into fresh LB medium containing 0.05 mg/mL carbenicillin, the cells were grown to OD_600_ = 0.6–0.8. Protein expression was induced with the addition 0.5 mM IPTG, at 37 °C.

After 3 h of expression, cells were harvested by centrifugation at 4 °C at 4000 rpm for 20 min. The pellet was resuspended in lysis buffer (50 mM Tris, 150 mM NaCl, 0.4 mM DTE, pH 7.5) supplemented with a protease inhibitor tablet (Roche, Basel, Switzerland) prior to usage. Sonication was performed for 6 × 15 s with 30 s pauses, on ice. Samples were centrifuged at 4 °C 5100 rpm for 20 min to remove cell debris. Using the high temperature resistance of ERD14, boiling the supernatant was performed for 10–15 min to precipitate globular proteins. The denatured proteins were removed by centrifugation at 4 °C, 16,000 rpm for 30 min. The supernatant was filtered through a 0.2 µm filter. The protein extract was purified with ÄKTAexplorer™ (GE Healthcare, Chicago, IL, USA) LC purification system. The first purification step was via buffer exchange on a HiPrep 26/10 Desalting column (GE Healthcare, Chicago, IL, USA). The sample was transferred to a 50 mM Tris buffer (pH 7.5).

Fractions containing ERD14 were purified on a Resource Q 6 mL (GE Healthcare, Chicago, IL, USA) anion exchange column. Elution was performed in 50 mM Tris buffer (pH 7.5) with a linear salt gradient up to 500 mM NaCl. Fractions selected from the chromatogram were checked by SDS PAGE on a 15% gel. Based on the gel image, the fractions containing pure protein were dialyzed into MQ water. Dialysis was performed at 4 °C O/N. The samples were lyophilized and stored at −20 °C until usage.

### 4.5. In Vitro Chaperone Assay

Thermal denaturation of citrate synthase (CS) was monitored in the presence of WT, FS, ∆Kc, and ∆S ERD variants, using CD spectroscopy and an aggregation assay.

Thermal induced transitions were monitored in CD experiments by recording the ellipticity at 220 nm, which is characteristic for the α-helix structural component of citrate synthase. Changes in ellipticity induced by the increasing temperature were correlated with the thermal unfolding of the enzyme.

In the aggregation assay, citrate synthase was incubated at elevated temperature in itself or in the presence of different ERD14 constructs (WT, FS, ∆Kc, or ∆S) The amount of denatured CS was determined by measuring the protein concentration before and after heat stress, after removing the aggregated proteins by centrifugation. Detailed description of the methods can be found in the Supplementary Methods.

### 4.6. Statistical Analysis

To assess the statistical significance of the observed differences, a large number of intra- and inter-experiment replicates were done. The survival rates showed minimal standard deviation within the same experiment, and only slightly larger deviation between experiments with a Gaussian distribution. Unpaired t-tests were used to quantify the significance of the differences between survival rates of the differently transformed bacteria.

### 4.7. CD Spectroscopy Measurements

Circular dichroism (CD) spectroscopy experiments were carried out on a Jasco J-810 instrument (Japan Spectroscopic Co., Tokyo, Japan) equipped with a Peltier-controlled thermostat. For secondary structure determination in 10 mM Na-phosphate, pH 7.4 buffer, and in the presence of 30% trifluoro-ethanol (TFE), a quartz cell of 10 μM pathlength was used with a protein concentration of ~5 mg/mL. Four scans were accumulated by using a scanning rate of 20 nm/min, a bandwidth of 1 nm, and a data integration time of 4 s.

SRCD spectra were recorded at the DISCO SRCD beamline of a SOLEIL Synchrotron (Gif-sur-Yvette, France). Experimental parameters were set by following the guidelines of Micsonai et al. [36]. Protein concentration was ~5 mg/mL in 10 mM Na-phosphate, pH 7.4, using a 12 µm CaF_2_ cell (Hellma GmbH, Müllcheim, Germany). At least 12 scans were accumulated in the 175–270 nm wavelength range at 1 nm steps with a lock-in time constant of 300 msec and integration time of 1200 msec. The spectra were treated with the CDTool package [37] (averaging, baseline subtraction, correction with camphorsulphonic acid (CSA), normalization). Secondary structure composition was analyzed by the BeStSel algorithm [38].

For the CD measurements, the protein concentrations were determined directly in the CD cuvettes by the absorbances at 205 and 214 nm. Measurements with the dialysis buffers were used as reference. The advantage of this method is that the extinction coefficients are large, even in the absence of aromatic residues, which is often the case for IDPs. Moreover, the actual CD samples are measured and thus any dilution errors or pathlength variations are taken into account. The extinction coefficients were calculated from the amino acid sequences on the BeStSel webserver (https://bestsel.elte.hu (accessed on various dates in April, 2021) following the protocol of Antis and Clore [39] and Kuipers and Gruppen [40] for 205 and 214 nm, respectively.

### 4.8. Quantification of the Contribution of ERD14 Motifs to Cell Protection

The protection of ERD14 and its various deletion and scrambled constructs of cells is primarily visualized through cell viability values measured after the applied stress (Supplementary Table S2). As viability before stress is taken as 100%, we observe that the viability of control cells (cells with an empty vector, or cells expressing GST, calpastatin, or scrambled ERD14) drops to 38.2% upon stress, whereas cells expressing ERD14 retain a viability of 74.5%. To derive the contributions of individual motifs or their combinations to this protective activity, we developed a % metric, which is 0% if addition of the motif keeps the activity of a given construct unchanged, whereas it is 100% if it raises its activity to the level of WT ERD14. As the background activity of a construct without the motif differs for the deletion and scrambling experiments, we have worked out a way to derive these two different values for the deletion and scrambled variants. For deletion constructs (e.g., ∆Kc in WT background), we took the viability (V) of the deletion construct after stress and assumed that adding back the deleted segment(s) would increase viability back to 100% (as it reconstitutes WT ERD14), thus the contribution of the segment is:

For example, viability of cells expressing ∆Kc is 0.549, which, upon “adding” Kc back, increases to 0.745. That is, the Kc segment increases the activity by 0.745–0.549, 53.99% of the potential maximum (cf. Table S2 and Figure 5C).

For scrambled mutants (e.g., Scr-Kc), we assumed that activity without the motif at 38.2% chaperone activity is 0% (because Full-Scr ERD14 exerts no protection), thus the measured actual V value represents the activity associated with the motif:

For example, viability of Scr-Kc is 0.397, i.e., Kc alone can add to the activity of Full-Scr ERD14 0.397–0.382, about 4.13% of the possible maximum (cf. Supplementary Table S2 and Figure 5C).

## Figures and Tables

**Table 1:** Effects of the deletions on the helical propensity of ERD14.

	∆Ka	∆Kb	∆Kc	∆Chp	∆H	∆S	Full-Scr
# of residues deleted	20	22	15	17	15	12	0
Change in the # of res. in helix	−10	−12	−18	−9	−7	−11	−29
DSR	3.0%	11.9%	19.6%	6.6%	9.8%	23.4%	35.6%

## Data Availability

The data presented in this study are available in the Supplementary Materials section.

## Supplementary Materials

The following are available online at https://www.mdpi.com/article/10.3390/ijms22126190/s1, Supplementary methods References [41,42,43,44,45,46,47,48,49,50,51]; Figure S1: Expression levels of ERD14 constructs in E. coli cells; Figure S2: Dose-effect correlation of ERD14; Figure S3: In vitro chaperone effect of different ERD14 constructs; Table S1: Sequences of ERD14 constructs and controls; Table S2: E. coli viability measurements; Table S3: Remaining residues in helix by deletion of different segments and the effects for the survival rates; Table S4: Melting temperature change of Citrate synthase in the presence of different ERD14 protein constructs.
