# Supplementary material for: Cellular Chaperone Function of Intrinsically Disordered Dehydrin ERD14
Source: Int J Mol Sci. 2021 Jun 8;22(12):6190. doi: 10.3390/ijms22126190 (PMC8230022; doi:10.3390/ijms22126190)
Supplement: Supplementary file 1 [file ijms-22-06190-s001.zip › ijms-1216138-supplementary.pdf]

Detailed Experimental Procedures

Constructs, recombinant protein, mutagenesis

Wild-type ERD14 was expressed from a previously prepared construct of ERD14 in a pET22b vector [30], which was cloned to contain no additional amino acids next to the original ERD14 sequence.

From wild-type ERD14, the conserved segments (Ka, S, Chp, Kb, H and Kc) were deleted individually (for all constructs, cf. Suppl. Table S2). Deletion mutants were prepared by PCR directly from the circular plasmid containing the wt-ERD14 gene. Primers were designed so that they align to the same sequence regions on opposite DNA strands and already incorporate the desired mutation/deletion. The correct DNA sequence was verified by DNA sequencing. Multiple deletion mutants DKab (deleting Ka and Kb), DKac, DKbc, DKcH, DKcS, and DKabc, were prepared in two or three consecutive steps.

For scrambled sequences (Full-Scr, Scr-Kc, Scr-S, Scr-Kabc, Scr-KcS, and Scr-KabcS), amino acids with the exception of the starting methionine (and the region specified) were scrambled in silico, followed by full cDNA synthesis. The constructs were cloned into a pET22b vector so that they did not contain any additional amino acids other than the scrambled sequences indicated in Suppl. Table S2.

For in-cell half-life measurements and quantification, the DNA fragment coding wild type ERD14 was amplified by Pfu Turbo HotStart DNA polymerase (Agilent) by PCR using ccgctcgagtgATGGCTGAGGAAATCAAGAATGTTCCTG forward and gaagatctTTCTTTATCTTTCTTCTCCTCCTCTACGG reverse primers and subcloned into the XhoI/BglII sites of expression vector pT7-FLAG 2 (Sigma). The C-terminal FLAG tagged fusion protein was expressed in the cytoplasm of BL21 star (DE3) cells under the control of the T7/lac promoter. pGEX-5X 1 vector was used to express GST or calpastatin protein in the same cell line as a control.

For the arabinose-inducible expression, the plasmid WT ERD14 - pBAd was constructed by cloning a 558-bp NheI-ERD14-KpnI fragment from plasmid pET22b into the NheI and KpnI sites of pBAD vector [76].

For pull-down analysis, WT ERD14 was subcloned into pAN4 vector (Avidity).

Scrambled ERD mutants

We used a multistep approach during the selection of the scrambled ERD14 mutant: (i) we generated 10,000 random sequences by using ERD14's amino acid composition, keeping the sequence set non redundant during the process; (ii) we used IUPred (long disorder prediction) to predict disorder propensity on all sequences and filtered out those that were significantly different from the disorder characteristics of the original ERD14 (different average disorder score or different disorder score variance); (iii) as the main reason of the scrambled mutant usage was to test the effect of the amino acid composition only, we used ANCHOR to predict potential interaction regions and exclude sequences with high confidence interaction regions; (iv) as the ERD14's amino acid composition was highly prone to randomly generate regions with high coiled-coil propensity we introduced a further filtering step, we used COILS to predict coiled-coil regions and exclude sequences with high coiled-coil propensity; (v) as a final step we used a charge distribution plot on the selected candidates (https://www.bioinformatics.nl/cgi-bin/emboss/charge) to visually filter out sequences with uneven charge distribution.

1. Generation of protein sequences (randomization)
For the segments to be mixed, 10 000 random protein sequences were generated by self-made Perl script. Starting metionin was always kept fixed.

In case of the full scrambled mutant the whole protein sequence was randomized, for the other constructs, sequences of the indicated segments were kept intact and sequences of the remaining parts were randomly mixed. E.g. in the ScrKc mutant, the sequence of the Kc segment was kept intact and the remaining sequences were scram-bled. For constructs with multiple regions intact, each intermittent sequence was treated separately, meaning that only the amino acids constituting that specific region were scrambled.

2. Coiled-coil prediction
To avoid introducing coiled-coil regions, the generated sequences were tested for coiled-coil content using COILS server (http://embnet.vital-it.ch/software/COILS_form.html; downloaded and run locally). Sequences containing predicted coiled-coils were excluded from the following analysis.

3. Disorder prediction
The remaining sequences were subjected to disorder prediction using IUPred pre-dictor (http://iupred.enzim.hu, with 'long disorder' as prediction option). We used not only the binary classification of IUPred (disordered above 0.5, ordered below 0.5), but also the residue level assigned disorder probabilities to calculate the average disorder probability and standard deviation for each sequence. We selected sequences that had a high average disorder with a low standard deviation, resulting in sequences that were predicted to be disordered throughout their entire length without regions of local ordered structures.

4. Filtering of interaction motifs
To avoid the involuntary introduction of interaction mofits, we used ANCHOR software (http://anchor.enzim.hu) to predict potential interaction regions and select sequences with the fewest potential molecular recognition motifs (with low probability as well) to avoid unwanted, aspecific interactions in vitro or in cell.

5. Post filtering methods
Filtered candidates were further analysed with the following tools:

ELM database: searching for interaction motifs based on sequence patterns to minimize the potential interaction capabilities (http://elm.eu.org)

Pfam database: to exclude sequences containing known structured or disordered domains (http://pfam.xfam.org)

Pasta 2.0 (http://protein.bio.unipd.it/pasta2/) and Aggrescan search (http://bioinf.uab.es/aggrescan/): to exclude sequences with aggregation prone regions.

Agadir analysis: to minimize the secondary structure elements within the sequence (http://agadir.crg.es)

Predict Protein: A metaserver with several different prediction modules (https://www.predictprotein.org) to filter out any possible unwanted structural elements.

6. Controlling charge distribution
As a final step we used a charge distribution plot on the selected candidates (https://www.bioinformatics.nl/cgi-bin/emboss/charge) to visually filter out sequences with uneven charge distribution

Cell viability assay

GST-, calpastatin- and ERD14 overexpressing BL21 (DE3) Star *E. coli* cells were grown in 5 ml LB medium containing 50 µg/ml carbenicillin overnight in 37°C under continuous shaking at 200 rpm. In the next day 50 µl aliquots were transferred into 5 ml LB medium containing 50 µg/ml carbenicillin and grown for 2 hours. Protein expression was induced with 0,5 mM IPTG for 3 hours. 0,5 ml aliquots were taken and stressed or not stressed before appropriate dilution into LB medium into the well of a 96 well plate. Samples were monitored in a BioTek Synergy Mx microplate reader for 12 hours at 37°C at 200 rpm shaking; reading of absorbance at 600 nm were taken every 2 minutes.

Viability was associated with the time it takes for the cells to reach an optical density of 0.55, which is about the half-maximal value they attain after long incubation periods (see curves of 10-, 30- and 90-times diluted cell suspension, Figure 2). Because this time varies linearly with the number of viable cells in suspension (Figure 2) that reflects the effect of stress, we could set up conditions appropriate for reproducibility and for providing a sufficient dynamic range for measuring the potential protective effect of ERD14 constructs. To this end, we selected 50◦C x 15 min stress for further studies on ERD14 effect. Finally, each measured cell viability value (survival rate) was corrected for the amount of protein expressed in the cell, as the protective effect of ERD14 constructs was shown to depend linearly on intracellular concentration (Suppl. Figure S2).

Thermal denaturation experiments by CD Spectroscopy

Circular dichroism (CD) spectroscopy experiments were carried out on a Jasco J-810 instrument (Japan Spectroscopic Co., Tokyo, Japan) equipped with a Peltier-controlled thermostat. For thermal denaturation studies of 1.3 µM citrate synthase was incubated alone or in the presence of various concentrations of WT (3.25 µM and 8.7 µM) and Full-Scr (FS) (8.7 µM) ERD14, ΔKc (8.7 µM) and ΔS (8.7 µM) in 10mM MES (pH = 6.5) 10 mM NaCl buffer. Fort he measurements a 1 mm pathlength cell was used. Thermal scans were carried out in the temperature range of 10–80 ◦C with a scanning rate of 2 ◦C/min. Thermally induced transitions were monitored by the ellipticity at 220 nm, which is characteristic for the α-helix structural component of citrate synthase. Experiments were carried out at least in duplicates. A two-state transition model described by [40] (Prot. Sci. 1995) showed a good fit to the denaturation curves and provided the melting temperature (Tm). We have to note that heating to 80 ◦C made the unfolding of citrate synthase irreversible, indicating that the results are rather limited to comparison of the samples than interpreting the absolute values.

In vitro chaperone measurement - Thermal denaturation experiments of citrate synthase

1.3 µM citrate synthase was incubated alone or in the presence of 8.7 µM of different ERD14 constructs (WT, FS, ΔKc or ΔS) in 10mM MES (pH = 6.5) 10 mM NaCl buffer. The protein solutions absorbances were measured at 310 nm, 205 nm and 214 nm in a Jasco … Spectrophotometer at 20°C, at least 2-2 parallels from each sample. After heat treatment was performed at 55°C for 15 min and denaturated CS fraction was removed by centrifugation at 16.000 rpm for 30 min, the supernatants absorbances were measured. CS fraction in solution after heat denaturation were obtained by the difference of initial and after treatment absorbances divided by the absorbance of CS solution before treatment. (Supplementary Figure S3B). Plotted data represent mean ± SEM results of at least 2 parallels for each construct. Significant differences compared to CS are labeled with asterisks (*).

Supplementary Tables

Supplementary Table S1. *Sequences of ERD14 constructs and controls*

(A) Deletion mutants of ERD14 are aligned to the sequence of WT ERD14, and the conserved segments (deleted in certain constructs) are highlighted in color. **(B)** Full sequences of scrambled mutants (highlighting in color the original motif kept, e.g. Kc segment kept in Scr-Kc, in green) are shown. It is to be noted that there is a practically unlimited number of possible “scrambled” sequences, i.e. all results shown pertain to the actual sequence (e.g. Full-Scr) selected. **(C)** Sequences of GST and calpastatin used as controls in the cell protection experiments.

(A) Deletion mutants

```
                                       Ka segment
ERD14 wt    MAEEIKNVPEQEVPKVATEESSAEVTDRGLFDFLGKKKDETKPEETPIASEFEQKVHISEPE
ΔKa         MAEEIKNVPEQEVPKVATEESSA--------------------EETPIASEFEQKVHISEPE
ΔKb         MAEEIKNVPEQEVPKVATEESSAEVTDRGLFDFLGKKKDETKPEETPIASEFEQKVHISEPE
ΔKc         MAEEIKNVPEQEVPKVATEESSAEVTDRGLFDFLGKKKDETKPEETPIASEFEQKVHISEPE
ΔS          MAEEIKNVPEQEVPKVATEESSAEVTDRGLFDFLGKKKDETKPEETPIASEFEQKVHISEPE
ΔChp        MAEEIKNVPEQEVPKVATEESSAEVTDRGLFDFLGKKKDETKPEETPIASEFEQKVHISEPE
ΔH          MAEEIKNVPEQEVPKVATEESSAEVTDRGLFDFLGKKKDETKPEETPIASEFEQKVHISEPE
ΔKac        MAEEIKNVPEQEVPKVATEESSA--------------------EETPIASEFEQKVHISEPE
ΔKbc        MAEEIKNVPEQEVPKVATEESSAEVTDRGLFDFLGKKKDETKPEETPIASEFEQKVHISEPE
ΔKabc       MAEEIKNVPEQEVPKVATEESSA--------------------EETPIASEFEQKVHISEPE
ΔKcS        MAEEIKNVPEQEVPKVATEESSAEVTDRGLFDFLGKKKDETKPEETPIASEFEQKVHISEPE
ΔKcH        MAEEIKNVPEQEVPKVATEESSAEVTDRGLFDFLGKKKDETKPEETPIASEFEQKVHISEPE
            ***********************                    *******************

                        S segment      Chp segment            Kb segment
ERD14 wt    PEVKHESLLEKLHRSDSSSSSSSEEEGSDGEKRKKKKEKKKPTTEVEVKEEEKKGFMEKLKE
ΔKa         PEVKHESLLEKLHRSDSSSSSSSEEEGSDGEKRKKKKEKKKPTTEVEVKEEEKKGFMEKLKE
ΔKb         PEVKHESLLEKLHRSDSSSSSSSEEEGSDGEKRKKKKEKKKPTTEVEVKE------------
ΔKc         PEVKHESLLEKLHRSDSSSSSSSEEEGSDGEKRKKKKEKKKPTTEVEVKEEEKKGFMEKLKE
ΔS          PEVKHESLLEK------------EEEGSDGEKRKKKKEKKKPTTEVEVKEEEKKGFMEKLKE
ΔChp        PEVKHESLLEKLHRSDSSSSSSSE-----------------PTTEVEVKEEEKKGFMEKLKE
ΔH          PEVKHESLLEKLHRSDSSSSSSSEEEGSDGEKRKKKKEKKKPTTEVEVKEEEKKGFMEKLKE
ΔKac        PEVKHESLLEKLHRSDSSSSSSSEEEGSDGEKRKKKKEKKKPTTEVEVKEEEKKGFMEKLKE
ΔKb         PEVKHESLLEKLHRSDSSSSSSSEEEGSDGEKRKKKKEKKKPTTEVEVKE------------
ΔKabc       PEVKHESLLEKLHRSDSSSSSSSEEEGSDGEKRKKKKEKKKPTTEVEVKE------------
ΔKcS        PEVKHESLLEK------------EEEGSDGEKRKKKKEKKKPTTEVEVKEEEKKGFMEKLKE
ΔKcH        PEVKHESLLEKLHRSDSSSSSSSEEEGSDGEKRKKKKEKKKPTTEVEVKEEEKKGFMEKLKE
            ***********            *                 *********

                        H region            Kc segment
ERD14 wt    KLPGHKKPEDGSAVAAAPVVVPPPVEEAHPVEKKGILEKIKEKLPGYHPKTTVEEEKKDKE
ΔKa         KLPGHKKPEDGSAVAAAPVVVPPPVEEAHPVEKKGILEKIKEKLPGYHPKTTVEEEKKDKE
ΔKb         ----------GSAVAAAPVVVPPPVEEAHPVEKKGILEKIKEKLPGYHPKTTVEEEKKDKE
ΔKc         KLPGHKKPEDGSAVAAAPVVVPPPVEEAHPV---------------YHPKTTVEEEKKDKE
ΔS          KLPGHKKPEDGSAVAAAPVVVPPPVEEAHPVEKKGILEKIKEKLPGYHPKTTVEEEKKDKE
ΔChp        KLPGHKKPEDGSAVAAAPVVVPPPVEEAHPVEKKGILEKIKEKLPGYHPKTTVEEEKKDKE
ΔH          KLPGHKKPED---------------EEAHPVEKKGILEKIKEKLPGYHPKTTVEEEKKDKE
ΔKac        KLPGHKKPEDGSAVAAAPVVVPPPVEEAHPV---------------YHPKTTVEEEKKDKE
ΔKbc        ----------GSAVAAAPVVVPPPVEEAHPV---------------YHPKTTVEEEKKDKE
ΔKabc       ----------GSAVAAAPVVVPPPVEEAHPV---------------YHPKTTVEEEKKDKE
ΔKcS        KLPGHKKPEDGSAVAAAPVVVPPPVEEAHPV---------------YHPKTTVEEEKKDKE
ΔKcH        KLPGHKKPED---------------EEAHPV---------------YHPKTTVEEEKKDKE
                                     ******               ***************
```

(B) Scrambled mutants

Full-Scr

MKTGKLPEETSAFEKSNESIVGETKEDKMEESPEPIVSKSKVEEQEEKKEKSTEALEVKPLP
EEYASRVEPGPHAEVFDGEKVRHGGEEPGFLPAHGKSSVKTEKESAIEVRHETTEPELVEQK

KLKPPIHKPKEDLEDVEHKEGVVDKKTLPVKDLSKIKSEKMKDSEAVPKAKSKEKEPKKFEA

Scr-Kc
MLDGLHEEHAADPSKDETSELFKTVGELSPRKFNKTDDKVSTRETEKKPEKSEKVEVEVEAS
KEEKSREKSAHVVKSVPGEEKHSKKSQDEIGKKEKEIPGEEVSPELKDGMPLPAEAEYKAVK
PEEVEVQPEGKEFEFTKPAPKSVEHSKIETAEKKGILEKIKEKLPGKKPVKESVLEEPPTH

Scr-S
MKEEEQGAREFIETLASEKTQESKEPILEVADKSPLVEDSLKTPKDEVEPIHPFGKNVAKPK
EFEEVVTHESELHRSDSSSSSSSKSEPEETPVSEEPIVPELKLMREEPEIEKTHKKPTVLPE
EKDAKGGEDPDVAFKHAKEKPGKGKKYKKEGKKKKKEELVPKEKKGVAEAKGEEVEKVTVH

Scr-SKc
MEFKAADKEIPEGEKHSEVIKASDPRIQEPFNLVKKETEEPFTKVEGEEKQPVTKVAPHEST
LESSDLVELEELHRSDSSSSSSSHPKESKVKKPEMPDEEAVGPEKKKGALFKEKKVGGEDVK
KPKAPVEGPSVEHEELEAETEAKKTRPKVEVEKKGILEKIKEKLPGEKDPKETKVYKHETE

Scr-SKabc

Scr-KabcS
MVKAESPQTPKEAEEVIENVESAEVTDRGLFDFLGKKKDETKPEEVESPITEFPEEESKPVS
EHKLLQKIAEHLHRSDSSSSSSSEGTEEKDEEKEPKKTKEKKERSKKVVGEEKKGFMEKLKE
KLPGHKKPEDAHVEPVEPVPAAAPPVAGVSVEKKGILEKIKEKLPGEKDPKETKVYKHETE

(C) Control proteins

GST
MSPILGYWKIKGLVQPTRLLLEYLEEKYEEHLYERDEGDKWRNKKFELGLEFPNLPYYIDGD
VKLTQSMAIIRYIADKHNMLGGCPKERAEISMLEGAVLDIRYGVSRIAYSKDFETLKVDFLS
KLPEMLKMFEDRLCHKTYLNGDHVTHPDFMLYDALDVVLYMDPMCLDAFPKLVCFKKRIEAI
PQIDKYLKSSKYIAWPLQGWQATFGGGDHPPKSDLIEGRGIPEFPGRLERPHRD

Calpastatin
MASHHHHHHGDPTETKAIPVSQQMEGPHLPNKKKHKKQAVKTEPEKKSQSTKLSVVHEKKSQ
EGKPKEHTEPKSLPKQASDTGSNDAHNKKAVSRSAEQQPSEKSTEPKTKPQDMISAGGESVA
GITAISGKPGDKKKEKKSLTPAVPVESKPDKPSGKSGMDAALDDLIDTLGGPEETEEENTTY
TGPEVSDPMSSTYIEELGKREVTIPPKYRELLAKKEGITGPPADSSKPIGPDDAIDALSSDF
TCGSPTAAGKKTEKEESTEVLKAQSAGTVRSAAPPQEKKRKVEKDTMSDQALEALSASLGTR
QAEPELDLRSIKEVDEAKAKEEKLEKCGEDDETIPSEYRLKPATDKDGKPLLPEPEEKPKPR
SESELIDELSEDFDRSECKEKPSKPTEKTEESKAAAPAHHHHHH

Supplementary Table S2 *E. coli viability measurements*

Survival rates of *E. coli* cells overexpressing a variety of constructs following heat stress (50°C x 15 min). The protein overexpressed in the cell, number of independent experiments, mean, SD and SEM values of cell viability are shown. Percent contribution of short binding motifs (Ka, Kb, Kc, Chp, H and S) and their combination against WT or scrambled (Scr) background are calculated as detailed in Experimental Procedures.

Protein name	Number of parallels	Mean	Standard Deviation	Standard Error of Mean	% effect against WT background (% incr. to max.)	% effect against Scr background (% incr. above min.)
No plasmid	9	0.262	é0.018	0.007		
Empty vector	9	0.368	0.025	0.009		
GST	30	0.384	0.114	0.021		
Calpastatin	18	0.387	0.064	0.015		
ERD14 wt	48	0.745	0.122	0.018		
Full-Scr	42	0.389	0.031	0.005		
ΔKa	24	0.715	0.11	0.022	8.26	
ΔKb	27	0.626	0.121	0.023	32.78	
ΔKc	24	0.549	0.077	0.016	53.99	
ΔKab	9	0.703	0.098	0.033	11.57	
ΔKac	9	0.588	0.042	0.014	43.25	
ΔKbc	15	0.598	0.072	0.019	40.50	
ΔKabc	24	0.589	0.139	0.029	42.98	
ΔChp	18	0.679	0.114	0.027	18.18	
ΔH	18	0.647	0.12	0.028	27.00	
ΔS	18	0.511	0.056	0.013	64.46	
ΔKcH	12	0.477	0.036	0.014	73.83	
ΔKcS	12	0.404	0.028	0.014	93.94	
Scr-Kc	18	0.397	0.060	0.014		4.13
Scr-KaKbKc	12	0.589	0.044	0.018		57.02
Scr-KcS	12	0.48	0.037	0.015		27.00
Scr-S	12	0.398	0.049	0.02		4.41
Scr-KabcS	12	0.572	0.032	0.016		52.34

Supplementary Table S3 *Remaining residues in helix by deletion of different segments and the effects for the survival rates*

Number of residues in helix of WT, single deletion mutants and Full-Scr construct of ERD14 protein according to the CD measurements conducted in the presence of 30% TFE and evaluating the measured spectra between 190-250 nm by BestSel[33,35] The measured helix contents show no correlation with the DSR values.

	**ERD14 WT**	**ΔKa**	**ΔKb**	**ΔKc**	**ΔChp**	**ΔH**	**ΔS**	**Full-Scr**
# of residues in helix	46	35	33	28	36	38	35	17
DSR	reference point	3.0%	11.9%	19.6%	6.6%	9.8%	23.4%	35.6%

Supplementary Table S4 *Melting temperature change of Citrate synthase in the presence of different ERD14 protein constructs.*

Melting temperature of CS was defined by thermal denaturation CD measurements. The experiments were carried out with 1.3 µM CS alone and in the presence of 3.25 µM ERD14 WT and 8.7 µM ERD14 WT, 8.7 µM Full-Scrambled (FS), 8.7 µM ΔKc and 8.7 µM ΔS construct (cf.: Suppl.Figure S3). Data represent mean ± SEM and the results of at least 2 parallels for each construct.

	1.3 µM CS	1.3 µM CS + 3.25 µM WT	1.3 µM CS + 8.6 µM WT	1.3 µM CS + 8.6 µM FS	1.3 µM CS + 8.6 µM ΔKc	1.3 µM CS + 8.6 µM ΔS
T_m (°C) ± SE	49.8 ± 0.1	52.4 ± 0.1	55.3 ± 0.4	50.1 ± 0.1	49.8 ± 0.1	51.3 ± 0.1

Supplementary Figures

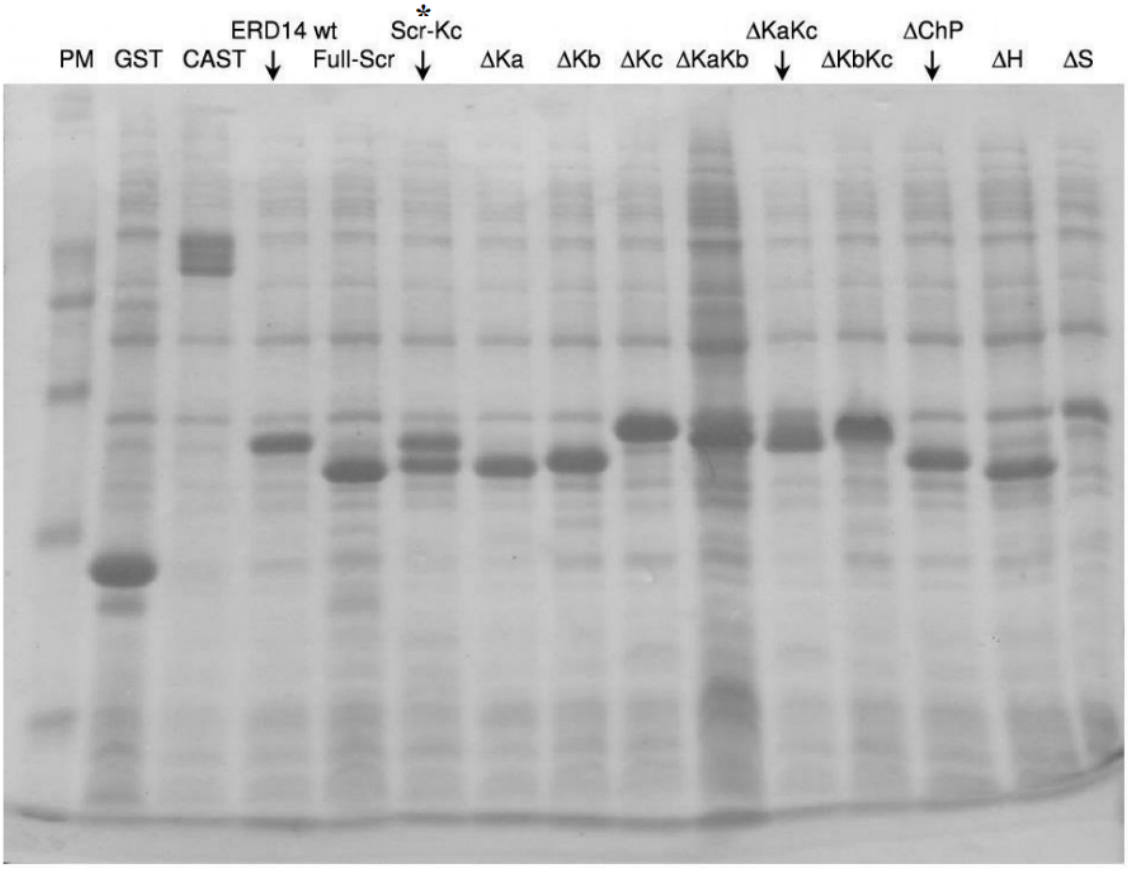

* Both bands attributable for Scr-Kc according to Mass Spectroscopy analysis.

Supplementary Figure S1 *Expression levels of ERD14 constructs in E. coli cells*

SDS-PAGE gel comparing the relative expression levels of different ERD14 constructs. Each measured survival rate was corrected for the molar amount of protein expressed within a given system to correct for concentration effects.

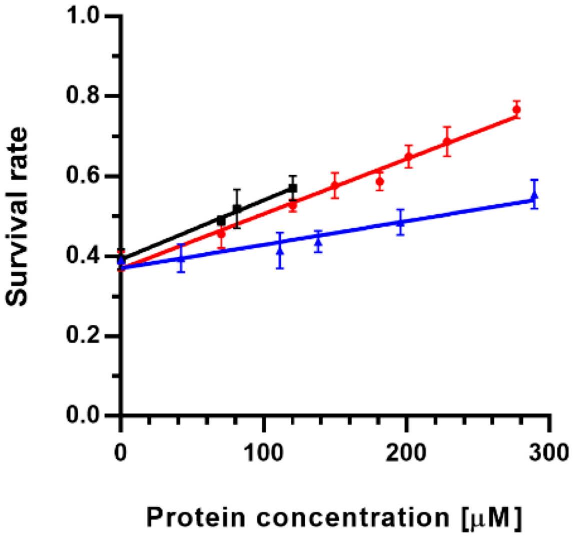

Supplementary Figure S2 *Dose-effect correlation of ERD14*

The protective effect increases with increasing concentration in the cell of ERD14 (red) or ΔKc (blue), measured by overexpressed FLAG-tagged proteins and determining their concentration by quantitative Western blotting. Linearity of response can also be observed when WT ERD14 is expressed by an arabinose inducible pBAD construct (black).

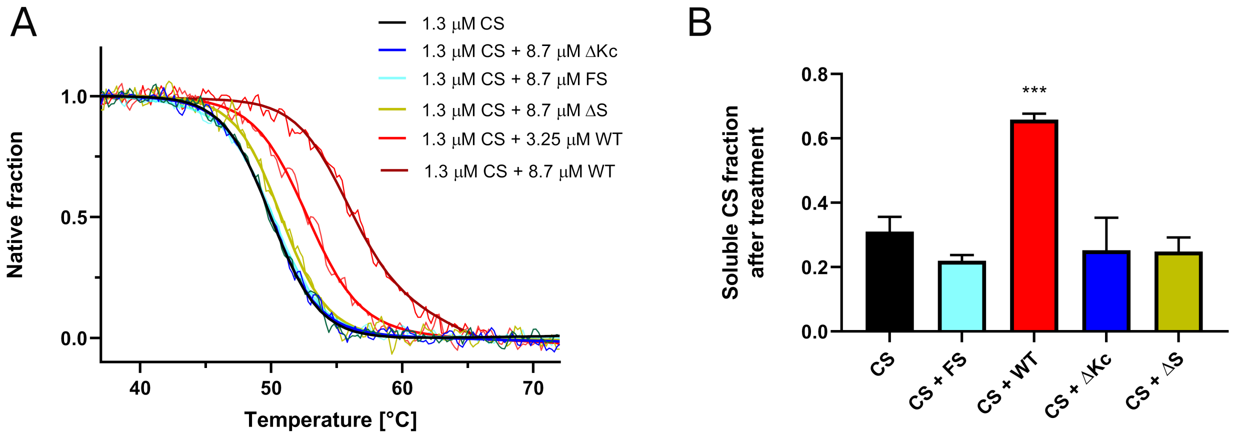

Supplementary Figure S3 *In vitro chaperone effect of different ERD14 constructs*

(A) Thermal denaturation measurement of 1.3 µM Citrate synthase (CS) alone (black) and in the presence of 3.25 µM ERD14 WT (lighter red) and 8.7 µM ERD14 WT (darker red), Full-Scrambled (FS) (teal), ΔKc (blue) and ΔS (ocher) construct. (B) CS fraction in solution after heat denaturation at 55°C for 15 mins alone (black) and in the presence of 6.5 times multiple molar excess of FS (teal), WT (red), ΔKc (lue) and ΔS (ochre) constructs. Data represent mean ± SEM and the results of at least 2 parallels for each construct. Significant differences compared to CS are labeled with asterisks (*).
